# Fairness in the use of limited resources during a pandemic

**DOI:** 10.1371/journal.pone.0270022

**Published:** 2022-06-16

**Authors:** Josef Schosser

**Affiliations:** School of Business, Economics and Information Systems, University of Passau, Passau, Germany; Beijing University of Posts and Telecommunications, CHINA

## Abstract

Capacity limitations are indispensable measures of social distancing in fighting COVID-19 and other pandemics. The paper at hand analyzes these restrictions from the viewpoint of fairness, understood as the possibility of equal access to the scarce resource. To this end, it employs the so-called El Farol Bar problem in conjunction with an adaptive learning approach. Particular emphasis is given to the distribution of information. Our results show that information is, indeed, central to the situation. Policy recommendations are derived.

## 1 Introduction

Capacity limitations are indispensable measures of social distancing in fighting pandemics [1–3]. Regarding COVID-19, the restrictions are almost ubiquitous (and include, among others, bars, beaches, gyms, houses of worship, libraries, movie theaters, museums, public transport services, restaurants, and retail stores). Where total attendance exceeds the capacity restriction, waiting costs or other inconveniences are the immediate consequence. In dealing with capacity limitations, individual decision-makers are often left to their own experiences. The resulting dynamics can lead to very uneven allocations [4]. However, support for the restrictions is essential to sustain in and eventually overcome a pandemic. For this reason, it is important that everyone can participate. The paper at hand analyzes capacity limitations from the viewpoint of “fairness”, understood as the possibility of equal access to the scarce resource. The framework of choice is game theory, more precisely, the so-called El Farol Bar problem. We emphasize the role of the information available and investigate its impact on individual decision processes and the resulting occupancy. Overall, the paper is the first to identify and treat the problem described. As with other problems caused by the pandemic, game theory offers a suitable conceptual lens [5, 6].

Arthur [7] introduced the El Farol Bar problem in the following way: Every Thursday evening, *M* people decide independently whether to go to a bar (the “El Farol” in Santa Fe, New Mexico). There is no collusion or prior communication among the possible attendees. Going to the bar is only enjoyable if it is not crowded, otherwise the individuals would prefer to stay home. The bar is crowded if more than *B* people show up, whereas it is not crowded, and thus enjoyable, if visitors are *B* or fewer. After each week, people are informed of the total attendance at the bar, which is subsequently used as history data by the individuals to improve decision-making in the upcoming weeks. Of interest is the dynamics of the numbers attending from week to week. A somewhat unusual feature of the above problem statement is the discontinuous transition from uncrowded to crowded when the capacity restriction is reached. While this may seem like an unrealistic assumption in “normal” times (at least for a bar), discontinuities and nonlinearities are prevalent in times of a pandemic and the associated social distancing measures.

The El Farol Bar problem can be seen as a prototypical model of distributed resource allocation. In essence, agents compete through adaptation for a finite resource [8]. A given agent’s utility depends on the number of other agents who choose to utilize the same resource. Therefore, the El Farol Bar problem belongs to both the class of congestion games [9] and the class of mean field games [10]. While the former emphasizes the nature of scarce resources, the latter deals with an individual agent’s interaction with the aggregate behavior of all other agents. Over the years, many variants of Arthur’s game have been developed [11]. Physicists and computer scientists work mainly in the context of the so-called minority game—an odd number of agents have to choose between two alternatives, and the ones that have chosen the least popular alternative (the “minority” action) receive the highest payoff [12–14]. Thereby, the information available to players is limited even further: They only observe which group was in the minority, not the number of players who chose that group. Applications of the minority game can be found in various problems of decentralized decision-making, including financial and energy markets, communication networks, and mobile edge computation [15–18].

We employ the setting of the original El Farol Bar problem, but use a differing learning rule. Arthur [7] equips players with individualized sets of ad hoc predictors (or response modes). In each round, players act on the predictor that was correct most frequently in the recent past. Given starting conditions and the fixed sets of predictors available to the participants, the overall dynamics are completely deterministic. In contrast, our article adopts the stochastic framework developed in Bell et al. [19] and Whitehead [20]. The associated adaptive learning rule exhibits several advantages: a clearer problem statement, a computationally simpler algorithm, and the ability to investigate the impact of different information distributions.

Most of the literature on the El Farol Bar problem and its derivatives is devoted to the search for decision rules that ensure low fluctuation (or volatility) in the use of the resource. The lower the volatility of aggregate action, the higher utilization on days with few visitors and the lower crowding costs on days with many visitors. Volatility can thus be seen as an inverse measure of global “efficiency”. This paper takes a different view. It emphasizes “fairness” (also known as “symmetry” or “impartiality”) in the sense of an “equal treatment of equals” [21, 22]. In our context, fairness requires that two identical agents end up with the same allocation [23]. The following results are derived: While incomplete historical information evokes an unfair allocation of resources, the provision of full information enables all to participate.

The remainder of the paper progresses as follows: Section 2 analyzes the El Farol Bar problem as a game in strategic form. In Section 3, we present our adaptive learning approach. Section 4 provides a discussion.

## 2 The El Farol Bar problem as a game in strategic form

In a natural first step, we consider the El Farol Bar problem as a one-shot simultaneous move game. For this purpose, we have to assume explicit payoffs for the different outcomes. The agents are supposed to be homogeneous. Let *b* denote the benefit an agent receives for attending an uncrowded bar. The payoff for attending a crowded bar equals −*b*. An agent that stays home obtains zero utility. As is well known, any game in strategic form with a finite number of players, and finitely many strategies per player, has at least one equilibrium in mixed strategies [24]. In our case, several equilibria exist. First of all, there are multiple equilibria in pure (i.e., “degenerate” mixed) strategies. In any one of them, exactly *B* agents attend the bar, while *M* − *B* agents stay home. Those agents visiting the bar have no incentive to stay home, where they would obtain zero payoffs. The agents that stay away have no incentive to attend the bar, since attendance by any one of them would suddenly cause crowding. Obviously, the outcome is efficient, but far from fair. Moreover, there is a unique symmetric equilibrium in mixed strategies. Thereby, all players randomize between the actions available and attend the bar with probability p=BM [7]. As the solution is symmetric, it generates a fair outcome. For several reasons, this game-theoretic investigation is not convincing. On the one hand, an equilibrium in mixed strategies requires the knowledge of the entire distribution of attendance. On the other hand, games in strategic form have no temporal component and, thus, do not allow for an analysis of evolving patterns. In the following, we make use of an adaptive learning rule that does not rely on the prediction of or the inference about other agents’ behavior. Instead, agents adapt their probability of attending over time based on the utilization data available to them.

## 3 The El Farol Bar problem in an adaptive learning framework

Each agent in our adaptive learning approach is characterized by a parameter that determines how often he/she attends the bar. This parameter changes in response to new information that comes the agent’s way. We do not require agents to make explicit predictions of their fellow agents’ decisions. Let *t* = 1, …, *T* denote a given time period. Suppose that agent *i* initially attends *p*_*i*_(1) percent of the time. He/she exploits successful strategies and goes more often (increases *p*_*i*_(*t*) slightly) if the bar is uncrowded, but prefers to go less often (to decrease *p*_*i*_(*t*)) if the bar is crowded. Moreover, *x*_*i*_(*t*) describes a Bernoulli random variable that assumes the value one with probability *p*_*i*_(*t*) and the value zero otherwise. Due to autonomous decision-making, total attendance in period *t* equals

$$\begin{array}{c} N\left(t\right)=\sum_{i=1}^{M}x_{i}\left(t\right). \end{array}$$

With regard to the information distribution, we distinguish two cases. Where the agents base their probability updates only on their own experiences, they utilize “partial information”. Where the agents have access to information about the use of the resource even when they do not themselves use it, they employ “full information”. In the case of partial information, learning takes place according to

$$\begin{array}{c} p_{i}\left(t+1\right)=\{\begin{array}{cc} 0, & \text{if}\;p_{i}\left(t\right)-\mu \left(N\left(t\right)-B\right)x_{i}\left(t\right)<0\\ 1, & \text{if}\;p_{i}\left(t\right)-\mu \left(N\left(t\right)-B\right)x_{i}\left(t\right)>1\\ p_{i}\left(t\right)-\mu \left(N\left(t\right)-B\right)x_{i}\left(t\right), & \text{otherwise.} \end{array} \end{array}$$

Thereby, the stepsize *μ* determines how much to change the attendance probability in response to the experiences gained. If the agent stays home, *p*_*i*_(*t* + 1) = *p*_*i*_(*t*) holds. The case of full information is described by the following dynamics:

$$\begin{array}{c} p_{i}\left(t+1\right)=\{\begin{array}{cc} 0, & \text{if}\;p_{i}\left(t\right)-\mu \left(N\left(t\right)-B\right)<0\\ 1, & \text{if}\;p_{i}\left(t\right)-\mu \left(N\left(t\right)-B\right)>1\\ p_{i}\left(t\right)-\mu \left(N\left(t\right)-B\right), & \text{otherwise.} \end{array} \end{array}$$

Here, an agent’s probability parameter is updated whether he/she has personally attended the bar or not. Either way, the agent’s original propensity to visit the bar, *p*_*i*_(1), is randomly initialized.

For simulation, we choose the following specification of parameters. We assume *M* = 100 agents. The maximum capacity is supposed to equal *B* = 60. The stepsize of adjustment obtains the value *μ* = 0.01. In order to observe the long-term dynamics of the system, we investigate *T* = 300 periods or iterations. Programming is done in Python.

**Fig 1:**
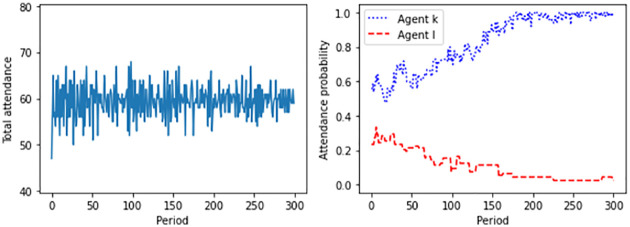
Total attendance and individual attendance probabilities of two representative agents in the case of partial information. Displayed are the results of a typical simulation run for *M* = 100 agents and *T* = 300 periods. The maximum capacity is assumed to equal *B* = 60. For the stepsize of adjustment, *μ* = 0.01 holds.

Our results are as follows. The observed behavior depends crucially on the nature of information available to the agents. Fig 1 displays one typical simulation run in the case of partial information. Here, the variance in the use of the resource slowly declines over the course of time. What is more, conditioning on own actions leads to heterogeneous beliefs. Consequently, the agents divide themselves into two groups. The attendance probability of one group of the agents, in Fig 1 represented by agent *k*, rises to very nearly one, indicating that they attend the bar every time. The remaining agents, in Fig 1 represented by agent *l*, use the resource less and less frequently, with their probability parameter approaching zero. Despite the stochastic nature of our adaptive learning rule, total attendance converges to a pure-strategy Nash equilibrium. This is consistent with recent findings on a more general class of learning processes [25].

**Fig 2:**
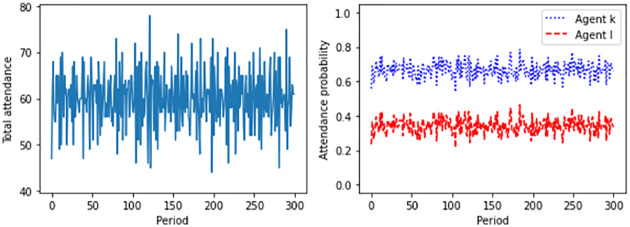
Total attendance and individual attendance probabilities of two representative agents in the case of full information. Displayed are the results of a typical simulation run for *M* = 100 agents and *T* = 300 periods. The maximum capacity is assumed to equal *B* = 60. For the stepsize of adjustment, *μ* = 0.01 holds.

Fig 2 shows the results of a typical simulation run in a scenario where full information prevails. In this case, the variance of aggregate attendance is somewhat higher and does not decline over time. The agents’ probability parameters fluctuate randomly around the initialized values. In fact, the attendance probabilities all increase or decrease simultaneously in response to the same signals. As a consequence, the population of users keeps changing in membership forever.

To examine the reliability of the identified patterns, we carry out a total of 1,000 simulation runs and record the attendance and the degree of fairness in the final period. Here, we use the variance in attendance probabilities,

$$\begin{array}{c} \frac{1}{M}\sum_{i=1}^{M}{\left(p_{i}\left(T\right)-\bar{p}\left(T\right)\right)}^{2}, \end{array}$$

where

$$\begin{array}{c} \bar{p}\left(T\right)=\frac{1}{M}\sum_{i=1}^{M}p_{i}\left(T\right), \end{array}$$

as a measure of the fairness associated with the outcome of a particular simulation run. The higher this quantity, the more unequal the access to the scarce resource. Table 1 provides descriptive statistics. Average attendance reaches the maximum capacity irrespective of the information available. However, there are clear differences in terms of participation levels: The case of full information proves to be more fair than that of partial information. This ranking holds both on average and for each individual simulation run.

**Table 1:** Descriptive statistics of total attendance and individual attendance probabilities in the final period depending on the nature of information available.

criterion	partial information	full information
average across attendances in the final period	59.566	59.931
variance across attendances in the final period	6.685	35.198
average across variances in final-period attendance probabilities	0.192778	0.061784
variance across variances in final-period attendance probabilities	0.000109	0.000040

*Note*. The variance in final-period attendance probabilities is interpreted as a quantitative measure of the fairness associated with the outcome of a particular simulation run. Displayed are the results of 1,000 simulation runs for *M* = 100 agents and *T* = 300 periods. The maximum capacity is assumed to equal *B* = 60. For the stepsize of adjustment, *μ* = 0.01 holds.

We performed sensitivity analysis to check the robustness of our results [26]. Changes in the capacity limit *B* leave the behavior of both total attendance and attendance probabilities qualitatively unchanged. Higher values of the stepsize *μ* accelerate the development, lower values have a slowing effect. Detailed results are available upon request.

## 4 Discussion

In practice, the case of partial information prevails. The agents cannot learn what happens at the bar (or other facility) of interest when they are absent. The only way to know if a resource is crowded is to get on the road and find out. Given this, the contribution of our adaptive learning model is twofold [27]. First, it demonstrates that partial information leads to an unfair resource allocation in the sense of a segmentation of the population. The probability parameter of each individual agent converges to either zero or one. Second, if fairness is given preference over efficiency, our model indicates a better way of organizing decentralized decision-making. Full information prevents the separation of a community; everyone can participate. To achieve this, the operators of capacity-constraint facilities should be encouraged to make historical utilization data publicly available (e.g., via their websites).

Do people really decide this way, based on probabilities and fixed adjustments? Of course not. But they do adapt their behavior if new information comes in, using some kind of inductive reasoning [7, 28]. In particular, the decision-making of visitors to social venues should be adequately captured by our simple calculus which induces low cognitive load. Moreover, our model disregards social relationships. To a certain extent, wide-ranging networks can substitute publicly available information. However, people are linked in varying degrees, either deliberately or due to external circumstances. Therefore, social relationships are not expected to solve the problem of fairness.

## Supporting information

S1 File Code: Fairness in the use of limited resources during a pandemic. The file contains the Python implementation.
(PDF)
