# Supplementary material for: Fairness in the use of limited resources during a pandemic
Source: PLoS One. 2022 Jun 16;17(6):e0270022. doi: 10.1371/journal.pone.0270022 (PMC9202943; doi:10.1371/journal.pone.0270022)
Supplement: S1 File — The file contains the Python implementation. (PDF) [file pone.0270022.s001.pdf]

Code: Fairness in the use of limited resources during a pandemic

In [1]:
```
# use of standard libraries
import numpy as np
import matplotlib.pyplot as plt
```

In [2]:
```
# generation of individual decisions
def perform_bernoulli(current_probs):
    generated = np.random.random(len(current_probs))
    r = np.where(generated <= current_probs, 1, 0)
    return r
```

In [3]:
```
# learning in case of partial information
def partial_information_learning(current_prob, observed_attendance, individual_decision):
    new_prob = current_prob - mue*(observed_attendance - B)*individual_decision
    conditions = [new_prob < 0, new_prob > 1]
    choices = [0.0, 1.0]
    new_prob = np.select(conditions, choices, default=new_prob)
    return new_prob
```

In [4]:
```
# learning in case of full information
def full_information_learning(current_prob, observed_attendance):
    new_prob = current_prob - mue*(observed_attendance - B)
    conditions = [new_prob < 0, new_prob > 1]
    choices = [0.0, 1.0]
    new_prob = np.select(conditions, choices, default=new_prob)
    return new_prob
```

A typical simulation run

In [5]:
```
# parameter specification
seed = 1902
num_agents = 100
num_iter = 300
B = 60
mue = 0.01
```

In [6]:
```
# data storage
total_attendance = np.zeros(num_iter)
individual_attendance = np.zeros(num_agents*num_iter).reshape((num_agents, num_iter))
probabilities = np.zeros(num_agents*(num_iter + 1)).reshape((num_agents, num_iter + 1))
```

In [7]:
```
print(total_attendance.shape)
print(individual_attendance.shape)
print(probabilities.shape)
```

```
(300,)
(100, 300)
(100, 301)
```

In [8]:
```
# random initialization
np.random.seed(seed)
initialization = np.random.random(num_agents)
probabilities[:, 0] = initialization
```

In [9]:
```
# time loop
for i in range(num_iter):
    individual_attendance[:, i] = perform_bernoulli(probabilities[:, i])
    total_attendance[i] = np.sum(individual_attendance[:, i])
    #probabilities[:, i+1] = partial_information_learning(probabilities[:, i], total_attendance[i], individual_attendance[:, i])
    probabilities[:, i+1] = full_information_learning(probabilities[:, i], total_attendance[i])
```

In [10]:
```
plt.figure(figsize=(10,3))
plt.subplot(1,2,1)
plt.plot(total_attendance)
plt.xlabel('Period')
plt.ylabel('Total attendance')
plt.ylim(39.5, 80.5)
plt.subplot(1,2,2)
plt.plot(probabilities[28, :], 'b:', label='Agent k')
plt.plot(probabilities[3, :], 'r--', label='Agent l')
plt.ylim(-0.05, 1.05)
plt.xlabel('Period')
plt.ylabel('Attendance probability')
plt.legend()
plt.savefig("C:/Users/HP/Desktop/Aktuelles/El Farol Bar problem/Latex/Fig2.pdf")
plt.show()
```

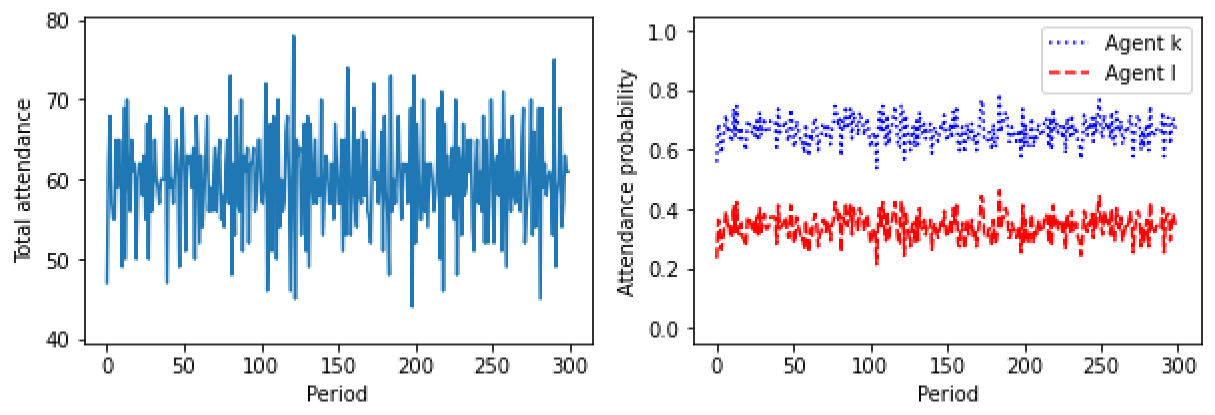

1,000 simulation run: descriptive statistics

In [11]:
```
# parameter specification
seed = 1902
num_runs = 1000
num_agents = 100
num_iter = 300
B = 60
mue = 0.01
```

In [12]:
```
# loop over simulation runs and time
np.random.seed(seed)
attendance_last_period = np.zeros(num_runs)
prop_variance_last_period = np.zeros(num_runs)
for i in range(num_runs):
    total_attendance_run = np.zeros(num_iter)
    individual_attendance_run = np.zeros(num_agents*num_iter).reshape((num_agents, num_iter))
    probabilities_run = np.zeros(num_agents*(num_iter + 1)).reshape((num_agents, num_iter + 1))
    initialization_run = np.random.random(num_agents)
    probabilities_run[:, 0] = initialization_run
    for j in range(num_iter):
        individual_attendance_run[:, j] = perform_bernoulli(probabilities_run[:, j])
        total_attendance_run[j] = np.sum(individual_attendance_run[:, j])
        #probabilities_run[:, j+1] = partial_information_learning(probabilities_run[:, j], total_attendance_run[j], individual_a
        probabilities_run[:, j+1] = full_information_learning(probabilities_run[:, j], total_attendance_run[j])
        attendance_last_period[i] = total_attendance_run[num_iter - 1]
        prop_variance_last_period[i] = np.var(probabilities_run[:, num_iter])
```

In [13]:
```
np.mean(attendance_last_period)
```

Out[13]:
```
59.931
```

In [14]:
```
np.var(attendance_last_period)
```

Out[14]:
```
35.198238999999994
```

In [15]:
```
np.mean(prop_variance_last_period)
```

Out[15]:
```
0.06178405841192978
```

In [16]:
```
np.var(prop_variance_last_period)
```

Out[16]:
```
4.093442557839699e-05
```

In [ ]:
```
# Storing the results of the case of partial information
prop_variance_last_period_partial = prop_variance_last_period
```

In [ ]:
```
# Storing the results of the case of full information
prop_variance_last_period_full = prop_variance_last_period
```

In [ ]:
```
# Ranking of the two cases
bigger = np.where(prop_variance_last_period_partial > prop_variance_last_period_full, 1, 0)
```

In [ ]:
```
# Counting the number of simulation runs where the case of partial information is less fair
np.sum(bigger)
```

In [ ]:
```

```
